# Neuroimaging Features of an Experimental Model of Extraparenchymal Neurocysticercosis

**DOI:** 10.1007/s11686-026-01393-z

**Published:** 2026-09-07

**Authors:** Sophia Rossi de Barros Almeida, Tatiane de Camargo Martins, Fátima Maria Caetano Caldeira, Vania Maria de Vasconcelos Machado, Marco Antônio Zanini, Pedro Tadao Hamamoto Filho

**Affiliations:** 1 Botucatu Medical School - Department of Neurosciences and Health, Universidade Estadual Paulista (Unesp), Botucatu, Brazil; 2 Faculty of Veterinary Medicine and Animal Science - Department of Surgery, Universidade de São Paulo, São Paulo, Brazil; 3 School of Veterinary Medicine and Animal Science Department of Veterinary Surgery and Animal Reproduction, Universidade Estadual Paulista (Unesp), Botucatu, Brazil

**Keywords:** Brain abnormalities, Experimental model, Taenia crassiceps, Neurocysticercosis

## Abstract

**Purpose:**

To retrospectively evaluate magnetic resonance images acquired from rats subjected to a murine model of extraparenchymal neurocysticercosis.

**Methods:**

The model consisted of injecting 50 *Taenia crassiceps* cysticerci into the cisterna magna of Wistar rats. To validate disease induction, magnetic resonance imaging was performed, focusing on the assessment of hydrocephalus and cysts distribution throughout the cerebrospinal fluid compartments. Ventricular dilation was quantified using the frontal horn ratio (FHR), calculated by dividing the maximal width of the frontal horns by the inner-table internal diameter.

**Results:**

Intracranial abnormalities were found 63.0% of the animals, predominantly ventricular dilation (> 90%), mainly involving the lateral ventricles. The most affected compartments were the cisterna magna (85.2%) and supratentorial basal cisterns (78.8%). Cysts were identified in the cerebral convexity in 10.6% of cases and in the subcutaneous tissue in 39.2% of infected animals. The mean FHR was 0.62 ± 0.13. Higher FHR values were associated with the presence of cysts in the CSF, but not in the subcutaneous tissue (*p* = 0.743).

**Conclusion:**

The translational relevance of this extraparenchymal NCC model is underscored by neuroimaging findings that closely resemble the human condition, providing a valuable platform for future mechanistic studies on pathogenesis and treatment development.

## Introduction

Neurocysticercosis (NCC) is one of the most common parasitic diseases affecting the central nervous system (CNS). Although its incidence has declined in some developing countries, particularly in Latin America, NCC remains endemic in regions with poor sanitation and is frequently diagnosed among Latin American immigrants in the United States and Europe [1–4].

When Taenia solium larvae reach the CNS, they may lodge either in the brain parenchyma or within cerebrospinal fluid (CSF) compartments, including the ventricles and subarachnoid space. Parenchymal NCC typically presents with seizures and headache. These symptoms can usually be managed clinically with antiepileptic and analgesic medications. The disease may resolve spontaneously or respond well to anthelmintic therapy, generally following a relatively benign course [5–7].

In contrast, extraparenchymal NCC responds less favorably to anthelmintic treatment and may lead to more severe complications, including vasculitis, impaired CSF circulation, and increased intracranial pressure related to hydrocephalus [8, 9]. Indeed, basal subarachnoid NCC is considered the most severe form of the disease, being associated with high rates of mortality and neurological disability. Treatment of extraparenchymal NCC remains challenging because cyst degeneration triggers a marked inflammatory response within the CSF compartments. As a result, hydrocephalus and intracranial hypertension may worsen after treatment initiation [10, 11]. Corticosteroids are commonly used to reduce inflammation, control symptoms, and prevent complications; however, they may also reduce the effectiveness of cysticidal agents [12]. Furthermore, current recommendations for the management of extraparenchymal NCC are based on studies with limited levels of evidence [13].

Experimental models may therefore provide valuable tools for preclinical testing of new therapeutic strategies. Several experimental models of NCC have been developed using different parasites (*T. solium*, *T. crassiceps*, and *Mesocestoides corti*) and host species, including monkeys, pigs, rats, and mice [14–19]. Among these, the rat model of extraparenchymal NCC induced by inoculation of *T. crassiceps* cysts into the subarachnoid space has demonstrated similarities to the human disease, including a chronic course, limited inflammatory response, and the development of hydrocephalus [20, 21].

In the present study, we evaluated magnetic resonance imaging (MRI) findings in rats inoculated with *T. crassiceps* cysts into the subarachnoid space in order to assess successful disease establishment, characterize patterns of cyst distribution throughout the CSF compartments, and investigate the development of hydrocephalus.

## Methods

This study was conducted at the Experimental Research Unit from Botucatu Medical School – São Paulo State University (UNESP). This is a retrospective study was based on the investigators’ database containing magnetic resonance imaging (MRI) records from 300 Wistar rats. These animals had been used in several studies involving the experimental model of extraparenchymal NCC. All study protocols were approved by the local Committee for Animal Care and Use (CEUA 1289/2019; 1318/2019; 1330/2019; 1377/2021; 1426/2023; 1427/2023; 1448/2024) and were conducted in accordance with ethical guidelines for animal experimentation.

### Experimental Procedures

Briefly, six-week-old Wistar rats (predominantly male) were anesthetized with an intraperitoneal injection of a ketamine (100 mg/mL) and xylazine (20 mg/mL) mixture at a dose of 0.1 mL/kg. A 1-cm skin incision was made along the midline of the occipitocervical region. The cisterna magna was then punctured using a 25-gauge needle, followed by the injection of 50 intact cysts (approximately 0.5 mm in diameter) suspended in 0.2 mL of 0.9% saline solution. The skin was closed with 4 − 0 mononylon sutures. A detailed description of the experimental procedures has been published previously [20].

### Imaging Acquisition and Analysis

Studies using this experimental model commonly employ MRI to confirm successful disease induction and to evaluate ventricular enlargement suggestive of hydrocephalus. MRI scans were obtained using a 0.25-T scanner (Esaote Vet-MR; Esaote, Santo André, São Paulo, Brazil) with the following parameters: slice thickness of 0.6 mm and T2-weighted gradient-echo acquisition (echo time, 5 ms; repetition time, 10 ms). MRI examinations were performed between one and six months after inoculation.

In the present study, we evaluated ventricular enlargement as well as enlargement of the subarachnoid space associated with cyst presence in the cerebral convexity, supratentorial basal cisterns, peritruncal cisterns, regions adjacent to the pineal gland, cisterna magna, spinal canal, and subcutaneous tissue. Due to the low power of our MR equipment, we assumed that enlarged cisterns corresponded to the presence of cysts, based on our empirical observations from MRI followed by euthanasia.

Ventricular enlargement was quantified using the frontal horn ratio (FHR), calculated as the ratio between the maximal width of the frontal horns and the inner-table-to-inner-table cranial diameter at the same level, according to the standard method used in humans. A FHR higher than 0.3 is indicative of hydrocephalus [22].

### Statistical Analysis

Categorical variables are presented as percentages. Continuous variables are expressed as mean ± standard deviation or median and interquartile range, as appropriate. Data distribution was assessed using the Shapiro–Wilk test. Differences between proportions were analyzed using the chi-square test or Fisher’s exact test. Parametric continuous variables were compared using Student’s t test, whereas nonparametric variables were analyzed using the Mann–Whitney test. Statistical significance was set at *p* < 0.05. Analyses were performed using SPSS Statistics for Windows, version 24.0 (IBM Corp., Armonk, NY, USA).

## Results

**Fig. 1:**
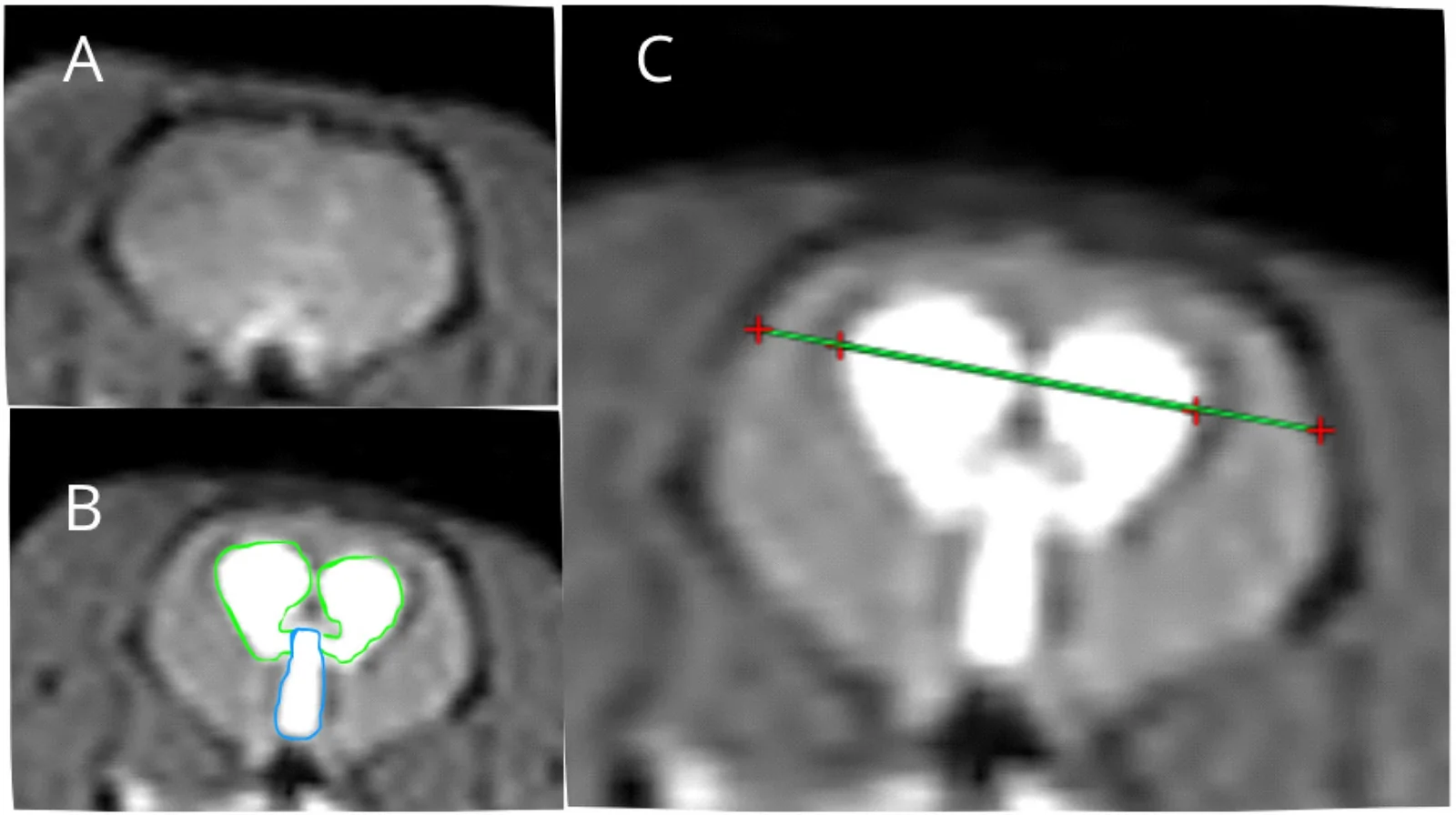
Magnetic resonance (MR) imaging scans obtained from rats subjected to the experimental model of extraparenchymal NCC. Normal transverse MRI image (**A**). Dilatation of the lateral ventricles is outlined in green, and dilatation of the third ventricle is outlined in blue (**B**). Hydrocephalus was quantified using the ratio between the maximal width of the lateral ventricles and the inner-table-to-inner-table diameter at the same level, frontal horn ratio (**C**)

Among the MRI records from 300 animals analyzed, 189 (63.0%) showed intracranial abnormalities, including ventricular enlargement and/or enlargement of the subarachnoid space. Ventricular enlargement was observed in more than 90% of the animals with intracranial abnormalities and predominantly involved dilation of the lateral ventricles (Fig. 1).

**Fig. 2:**
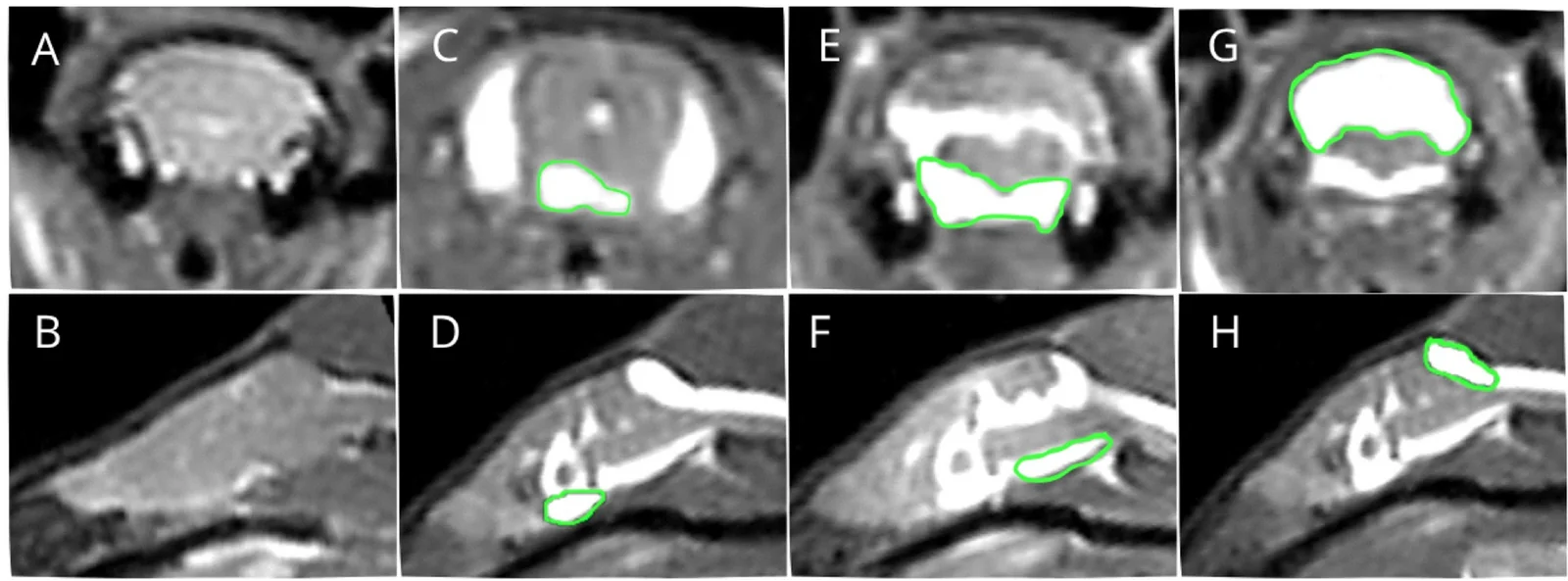
MR scans obtained from rats subjected to the experimental model of extraparenchymal NCC. Normal transverse (**A**) and sagittal (**B**) MRI images. Enlarged subarachnoid spaces suggestive of cyst presence are outlined in green. Supratentorial basal cysts (**C**, **D**), peritruncal cysts located anterior to the brainstem (**E**, **F**), and cysts within the cisterna magna (**G**, **H**)

**Fig. 3:**
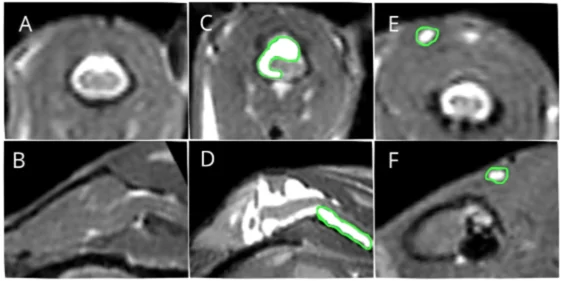
MR scans obtained from rats subjected to the experimental model of extraparenchymal NCC. Normal transverse (**A**) and sagittal (**B**) MRI images. Enlarged subarachnoid spaces suggestive of cyst presence are outlined in green. Cysts within the spinal canal located posteriorly and laterally (**C**), and anterior to the spinal cord (**D**). Subcutaneous cysts were also detected (**E**, **F**)

**Fig. 4:**
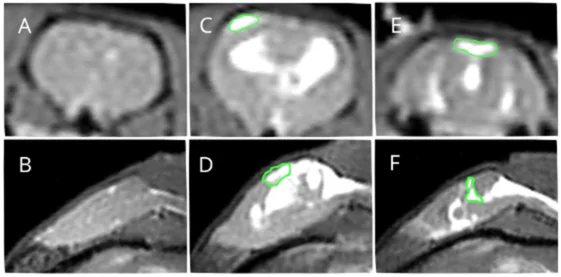
MR scans obtained from rats subjected to the experimental model of extraparenchymal NCC. Normal transverse (**A**) and sagittal (**B**) MRI images. Enlarged subarachnoid spaces suggestive of cyst presence are outlined in green. Abnormal enlargement of the subarachnoid space suggestive of cysts can be observed along the cerebral convexity (**C**, **D**) and near the pineal region (**E**, **F**)

The cisterna magna was the most frequently affected CSF compartment (85.2%), i.e., with increased dilation suggestive of the presence of cysts, followed by the supratentorial basal cisterns (78.8%). Cysts located along the cerebral convexity were identified in 10.6% of cases. Subcutaneous cysts were detected in 39.2% of infected animals (Table 1; Figs. 2, 3 and 4).

**Table 1:** Proportion of ventricle enlargement and affected sites among infected animals

Compartments	*n* (%)
Ventricles	
Lateral ventricles	178 (94.2)
III ventricle	176 (93.1)
IV ventricle	167 (93.1)
Subarachnoid space	
Cisterna magna	161 (85.2)
Supratentorial basal cisterns	149 (78.8)
Spinal canal	136 (72.0)
Peritruncal	108 (57.1)
Pineal region	82 (43.3)
Cerebral convexity	20 (10.6)
Subcutaneous	74 (39.2)

The frontal horn ratio (FHR) ranged from 0.28 to 0.92, with a mean value of 0.62 ± 0.13. The presence of cysts in each CSF compartment was associated with higher FHR values (Table 2), whereas the presence of subcutaneous cysts was not associated with differences in FHR (0.622 vs. 0.616, *p* = 0.743).

**Table 2:** Differences on FHR (mean ± standard deviation) according to the presence of cysts in CSF compartments (i.e., enlarged cisterns)

Site	Cysts *N*; mean ± sd	No cysts *N*; mean ± sd	Mean difference (95% CI)	*p*-value
Cisterna magna	155; 0.629 ± 0.126	23; 0.546 ± 0.133	0.083 (0.027–0.139)	0.004
Spinal canal	133; 0.635 ± 0.132	45; 0.567 ± 0.101	0.068 (0.025–0.111)	0.002
Supratentorial basal	149; 0.635 ± 0.127	29; 0.529 ± 0.108	0.106 (0.057–0.156)	< 0.001
Peritruncal	104; 0.641 ± 0.137	74; 0.585 ± 0.111	0.056 (0.018–0.094)	0.004
Pineal region	100; 0.672 ± 0.124	78; 0.576 ± 0.118	0.096 (0.060–0.132)	< 0.001
Convexity	20; 0.743 ± 0.133	158; 0.602 ± 0.120	0.141 (0.084–0.198)	< 0.001

N: number of animals; sd: standard deviation; CI: confidence interval

Eleven animals (5.8%) were considered infected despite the absence of ventricular enlargement. In these animals, cysts were more commonly identified in the subcutaneous tissue (7/11) and in the cisterna magna (6/11). Finally, subcutaneous cysts were detected in 39.2% of intracranially infected animals and in 30.2% of noninfected animals, with no significant difference between groups (*p* = 0.138).

## Discussion

Neurocysticercosis (NCC) remains endemic in several regions worldwide as a neglected disease, despite being potentially eradicable [23]. Although substantial progress has been made regarding the diagnosis and treatment of NCC, important gaps remain in the understanding of extraparenchymal disease, particularly with respect to treatment efficacy [24, 25]. Extraparenchymal cysts often respond poorly to anthelmintic therapy because of interindividual variability in albendazole metabolite concentrations within the CSF, the larger volume of the parasites, and the concomitant use of corticosteroids, which are administered to mitigate the inflammatory response triggered by parasite destruction [26–28]. Surgical cyst removal may reduce the need for prolonged anthelmintic treatment and provide faster clinical improvement [29, 30]. However, multiple surgical procedures are often required, and, as with any neurosurgical intervention, there is a risk of postoperative neurological deficits.

Hydrocephalus is a common complication of extraparenchymal NCC and results from both inflammation and mechanical obstruction of CSF flow [31, 32]. Management of hydrocephalus in NCC remains challenging. Many patients require repeated shunt revision surgeries and, although endoscopic approaches offer important advantages, not all patients are suitable candidates for endoscopic third ventriculostomy, particularly those with diffuse subarachnoid inflammation or adhesive arachnoiditis. Moreover, hydrocephalus in NCC is associated with a higher risk of unfavorable outcomes [11, 33, 34].

Experimental models of NCC are therefore essential to improve understanding of disease pathophysiology and to evaluate novel therapeutic strategies, including the development of vaccines [19]. Hydrocephalus has been reported in several experimental NCC models. The Cysticercosis Working Group in Peru developed an important model based on intracranial injection of activated *T. solium* oncospheres in rats, in which ventricular enlargement was observed in animals harboring intraventricular cysts [15]. This model is particularly valuable because it uses the same parasite species responsible for human disease. However, obtaining oncospheres from patients with taeniasis is not always feasible.

*T. crassiceps* has long been used as an experimental model of cysticercosis through intraperitoneal infection. Earlier attempts to establish intracranial infection with *T. crassiceps* were initially considered unsuitable for NCC research because the parasites displace rather than invade brain tissue [17]. Nevertheless, tissue displacement is precisely one of the main pathological features of extraparenchymal NCC. Mattos-Silva et al. inoculated *T. crassiceps* cysts intracranially and demonstrated that some cysts eventually migrated into the ventricles, leading to asymmetric ventricular enlargement [17]. Subsequently, the same group standardized an intraventricular inoculation model, which provided important insights into local and systemic cytokine responses, as well as parasite energy metabolism following cysticidal treatment [33].

In our model, cysts are directly injected into the subarachnoid space, resulting in obstruction of CSF outflow from the fourth ventricle. Inflammation along the ependymal lining has also been observed, suggesting that both mechanical obstruction and inflammation contribute to the development of hydrocephalus [34]. Nevertheless, as demonstrated in subsequent studies, the inflammatory response appears to be attenuated, probably due to the action of regulatory T cells (Tregs). More intense inflammation becomes evident after anthelmintic treatment in the absence of concomitant corticosteroid therapy [35].

The present neuroimaging findings further support the similarities between this experimental model and human extraparenchymal NCC. For example, Bazan et al. reported that 86.1% of patients with extraparenchymal NCC presented cysts within the posterior fossa cisterns [36]. In our model, this distribution corresponds to the enlargement observed in the cisterna magna (85.2%) and peritruncal cisterns (57.1%). This finding is expected, given that cysts are injected directly into this compartment.

Interestingly, cysts were also identified within the ventricles and supratentorial cisterns, indicating that they can migrate across different CSF compartments, as has been described in humans. Similar observations were also reported in the adaptation of this model proposed by Espinosa-Cerón et al. [21]

Spinal NCC is an uncommon manifestation of the disease and a rare cause of myelopathy. Motor deficits and back pain are the most frequent symptoms in cases of intradural or intramedullary cysticercosis, and the radiological presentation resembles that observed in cerebral infection [37, 38]. Subarachnoid spinal cysts (intradural and extramedullary), in addition to causing motor and pain symptoms, are also commonly associated with hydrocephalus, [37, 39] further reinforcing the similarities between spinal disease and our model. In fact, this model may provide useful insights into spinal cord compression in NCC.

One limitation of using a parasite species different from that responsible for human disease is the uncertainty regarding the direct translational applicability of the findings, since species-specific host responses may not be fully reproduced [19 ]. Nevertheless, this model has consistently demonstrated close similarities to human extraparenchymal NCC, and the present neuroimaging findings provide additional supportive evidence despite the limitations inherent to the use of a low-field open MRI scanner. Using low-power equipment neither allowed us to quantify the number of cysts nor to accurately distinguish enlarged cisterns from the presence of cysts (and, in humans, these two findings do not always correspond). Nevertheless, our findings are comparable to those reported in studies using a 7-T MRI system, [21] although our model employed a higher parasite burden (50 vs. 30 cysts), which may explain the greater disease severity observed, particularly with respect to hydrocephalus.

In conclusion, the neuroimaging findings observed in this experimental model of extraparenchymal NCC closely resemble those of human disease, supporting its utility as a relevant platform for future investigations into disease pathophysiology and the development of new therapeutic approaches.

## Data Availability

No datasets were generated or analysed during the current study.
